# The complete mitochondrial genome sequence of the *Metschnikowia bicuspidata* (Metschnikoff, 1884), an emerging pathogen of ‘milky disease’ in Chinese mitten crab

**DOI:** 10.1080/23802359.2022.2097025

**Published:** 2022-07-12

**Authors:** Jiannan Liu, Jieying Yu, Senting Pu, Xinran Shi, Yihao Li, Xiaoran Zhao, Shigen Ye

**Affiliations:** Aquatic Animal Hospital of Dalian Ocean University, Dalian Ocean University, Dalian, China

**Keywords:** *Metschnikowia bicuspidata*, mitochondrial genome, *Eriocheir sinensis*

## Abstract

The outbreak of milky disease of Chinese mitten crab caused by *M. bicuspidata* seriously restricted the development of the crab industry. In this study, the mitochondrial genome sequence of *M. bicuspidata* was assembled, annotated, and further analyzed. The results indicated that the complete mitochondrial genome of *M. bicuspidata* was 75,095 bp, which contained two rRNAs, 23 tRNAs, and 13 protein-coding genes. The phylogenetic tree of 13 yeasts based on the complete mitochondrial genome was constructed which showed that *M. bicuspidata* (accession number OK514652) and *M. bicuspidata* (accession number MW147605.1) were clustered in a clade. To sum up, our research results would further provide essential data for the systematics and evolution study of *M. bicuspidata*.

Chinese mitten crab (*Eriocheir sinensis*) belongs to the phylum Arthropoda, class Crustacea, order Decapoda, family Grapsidae, and genus *Eriocheir*. It is an important economic crab in China (Fu et al. 2017). In 2018, the total production of *E. sinensis* reached 692,723 tonnes in China, with an output value of 9,386,536 thousand dollars (http://www.fao.org/fishery/statistics, 2018). At present, the breeding areas of *E. sinensis* in China are mainly concentrated in Jiangsu, Hubei, Anhui, and Liaoning provinces. However, with the continuous expansion of the scale and density of crab farming, the disease has been one of the most important limitations of the sustainable development of the industry (Sui et al. 2012; Ding et al. 2015, 2016; Bao et al. 2021). In 2019, a pathogenic yeast was isolated from diseased Chinese mitten crab in many farms of Panjin City, Liaoning Province, China (121°25′–122°31′E, 40°39′–41°27′N), which was identified as *Metschnikowia bicuspidata* (Metschnikoff, 1884) belongs to family Metschnikowiaceae, genus *Metschnikowia* (Ma et al. 2020, 2022; Bao et al. 2021). However, the mitochondrial genome sequence of *M. bicuspidata* was still in the blank.

The specimen of *M. bicuspidata* (Number: 2EJM001) was stored in the Aquatic Animal Hospital of Dalian Ocean University, Dalian Ocean University, Dalian, China. We constructed the library with an average length of 350 bp using the NexteraXT DNA Libraries Preparation Kit (Illumina, San Diego, CA, USA) after the libraries were sequenced on Illumina Novaseq 6000 platform. The raw data totaled 2.84 G, and the clean data totaled 2.83 G after quality control processing and yielding a 1231-fold depth of coverage of the mitochondrial genome. The GC content of the clean data was 46.5%, the Q20 value was 97.90%, and the Q30 value was 93.96% which indicated that the quality of the mitochondrial genome sequencing and assembly results was very high. High-quality reads were assembled into the mitochondrial genome using *de novo* assembler SPAdes v.3.11.0 software (Bankevich et al. 2012). Finally, we annotated the assembled complete mitochondria genome by MITOS (Bernt et al. 2013). The sequence of *M. bicuspidata* mitochondrial genome was deposited in GenBank (accession number OK514652). Phylogenetic relationships between *M. bicuspidata* and other 12 yeasts were analyzed according to the whole mitochondrial genome from GenBank using Maximum Likelihood methods by MEGA 7.0.

All analytical results indicated that the complete mitochondrial genome of *M. bicuspidata* was 75,095 bp. Its nucleotide composition was as follows: A, 39.1%; C, 11.8%; G, 13.5%; T, 35.6%, with a high AT content of 74.7% which was higher than other kinds of yeast, such as *Sporobolomyces* sp and *Cystobasidium* sp (Huang et al. 2020; Liu and Wang 2020). The circular mitogenome of this yeast includes two rRNAs (16S rRNA and 12S rRNA), 23 tRNAs (tRNA-Ser, tRNA-Thr, tRNA-Val, tRNA-Cys, tRNA-Leu, tRNA-Glu, tRNA-His, tRNA-Met, tRNA-Tyr, tRNA-Asp, tRNA-Arg, tRNA-Phe, tRNA-Gly, tRNA-Asn, tRNA-Pro, tRNA-Lys, tRNA-Ile, tRNA-Gln, tRNA-Ala, and tRNA-Sec) and 13 protein-coding genes (PCGs). From the 13 PCGs of *M. bicuspidata* mitogenome, nine PCGs (*nad1*, *nad4*, *nad6*, *atp6*, *atp9*, *cob*, *cox1*, *cox2*, and *cox3*) were located on the heavy strand and four PCGs (*nad2*, *nad3*, *nad4L*, and *nad5*) on the light strand. Among these PCGs, three genes (*nad3*, *nad4L*, and *nad2*) used the start codon ATT, 4 genes (*nad2*, *atp6*, *atp9*, and *cox2*) used the start codon ATA, and the other genes (*cob*, *cox1*, *cox3*, *nad1*, *nad4*, and *nad6*) used the start codon ATG. Besides, except that the stop codon of *nad2* was TAG, the stop codon of other PCGs (*cob*, *cox1*, *cox2*, *cox3*, *nad1*, *nad3*, *nad4*, *nad4L*, *nad5*, *nad6*, *atp6*, and *atp9*) was TAA.

The resulting phylogenetic tree showed that there were two main clades, Metschnikowiaceae and Sporidiobolaceae. Twelve yeasts species were clustered together within Metschnikowiaceae; *Metschnikowia bicuspidata* (accession number OK514652) and *Metschnikowia bicuspidata* (accession number MW147605.1) were clustered in a clade (Figure 1). In conclusion, the mitochondria of *M. bicuspidata* reported in this study provided more essential information about the yeast, which will help our understanding of *M. bicuspidata* better. Further, the information will do good to develop the rapid detection methods which will favor the control of the 'milky disease' in Chinese mitten crab.

**Figure 1.**
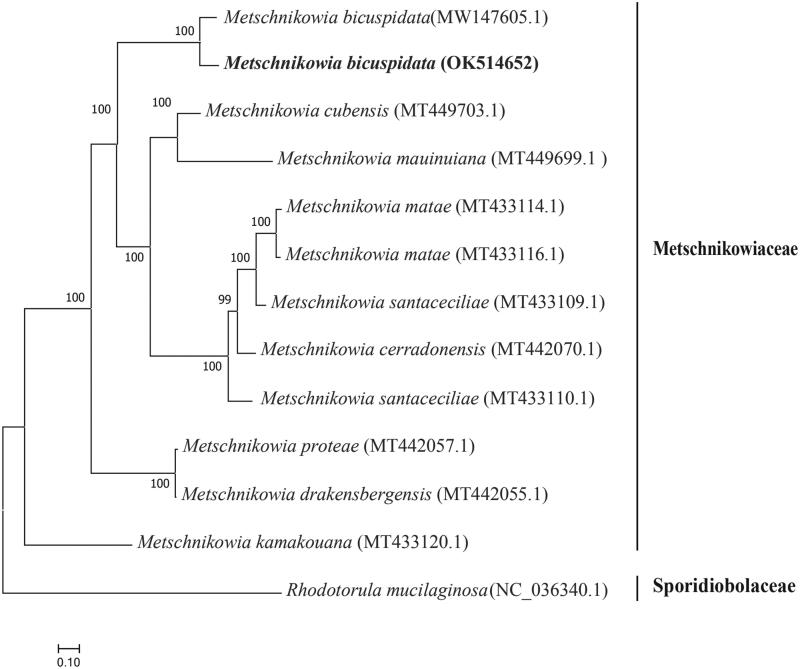
The phylogenetic tree was inferred using the maximum-likelihood method based on the complete mitochondrial genome of 13 yeasts. A total of 1000 bootstrap replicates were computed, and the bootstrap support values are shown at the branches.

## Storage location of the specimen or its DNA

The specimen of *M. bicuspidata* (Number: 2EJM001) was stored in the Aquatic Animal Hospital of Dalian Ocean University, Dalian Ocean University, Dalian, China. And the e-mail of Shigen Ye, the person in charge of the collection, is shgye@dlou.edu.cn.

## Data Availability

The genome sequence data that support the findings of this study are openly available in GenBank of NCBI at [https://www.ncbi.nlm.nih.gov/nuccore/OK514652] under the accession no. OK514652. The associated BioProject accession number of the genome is PRJNA771255 [https://www.ncbi.nlm.nih.gov/bioproject/PRJNA771255]. The associated BioSample accession number of the genome is SAMN22266430 [https://www.ncbi.nlm.nih.gov/biosample/?term=SAMN22266430]. The associated SRA is SRR16328191 [https://www.ncbi.nlm.nih.gov/sra/SRR16328191].
